# Supplementary material for: Identification of Freezing-Responsive microRNAs and Their Targets in Chinese Jujube by Small RNA and Degradome Sequencing
Source: Int J Mol Sci. 2026 Sep 4;27(17):7912. doi: 10.3390/ijms27177912 (PMC13566890; doi:10.3390/ijms27177912)
Supplement: Supplementary file 1 [file ijms-27-07912-s001.zip › Supplementary Material S2/novel_miR_47.pdf]

Provisional ID	:	novel_miR_47
Score total	:	0.4
Score for star read(s)	:	-1.3
Score for read counts	:	0
Score for mfe	:	0.1
Score for randfold	:	1.6
Score for cons. seed	:	
Total read count	:	105
Mature read count	:	105
Loop read count	:	0
Star read count	:	0

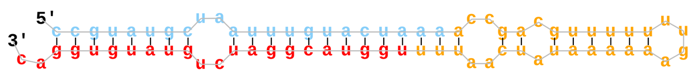

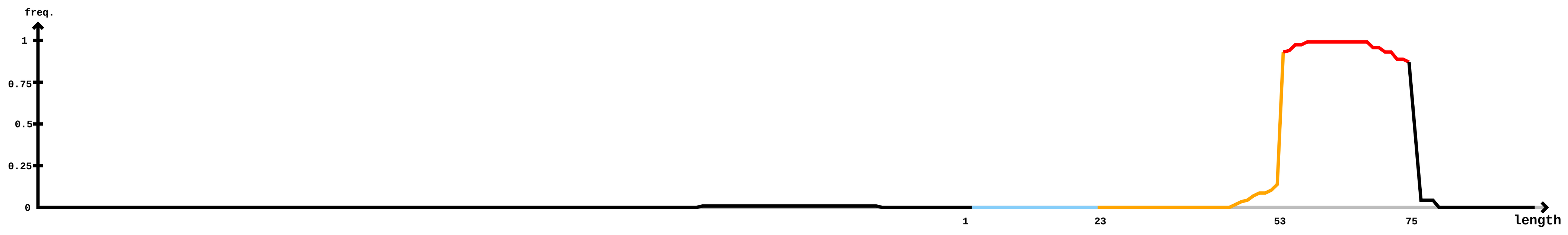

Star Mature

5'- ugugugugug[illegible]ugugggggugcggaccgucgu ccguaugcuaauuuguacuaaa accgacguuuuuuugaaaaaauaucaauuu ugguacggaucuguauguggac gauccauaccauagaaauauau -3' exp

reads (sequence)	reads	mm	sample
guguguguguguguguguguguguguguguCugg	1	1	JCK-3
aucGauuuugguacggaucugua	1	1	JCK-2
aucGauuuugguacggaucugua	1	1	JCK-1
ucGauuuugguacggaucuguaug	2	1	JCK-2
cGauuuugguacggaucuguaug	1	1	LT-1
Gauuuugguacggaucuguaugug	1	1	JCK-2
Gauuuugguacggaucuguaugug	1	1	JCK-3
aauuuugguacggaucuguaugug	1	0	JCK-3
auuuugguacggaucugua	2	0	LT-2
uuugguacggaucuguaugug	1	0	JCK-2
uuugguacggaucuguaugug	1	0	JCK-3
uugguacggaucuguaugugAac	1	1	JCK-1
uugguacggaucuguaugugAac	2	1	JCK-2
uugguacggaucuguaugugAac	1	1	JCK-3
ugguacggaucuguauguggC	2	1	LT-3
ugguacggaucuguaugugAac	9	1	JT-1
ugguacggaucuguauguggac	32	0	LCK-2
ugguacggaucuguaugugAac	8	1	LT-2
ugguacggaucuguaugugAac	8	1	LT-1
ugguacggaucuguaugugAac	5	1	LT-3
ugguacggaucuguauguggac	27	0	LCK-1
ugguacggaucuguNuguggac	1	1	LCK-1
gguacggaucuguaugugAac	1	1	LT-1
guacggaucuguauguggacgauc	3	0	JCK-2
guacggaucuguauguggacgauc	1	0	JCK-3
acggaucuguaugugAac	1	1	LT-3
acggaucuguauguggacgaAc	1	1	LT-1
